# Supplementary material for: Physician behavior for “invisible” treatment; Korean herbal medicine doctor’s treatment covered by auto insurance
Source: PLoS One. 2026 Jun 3;21(6):e0350512. doi: 10.1371/journal.pone.0350512 (PMC13232845; doi:10.1371/journal.pone.0350512)
Supplement: Attachment — Submitted filename: PLOS one reviewers comments.pdf [file pone.0350512.s002.pdf]

**Summary of the study**

In this study, the author hypothesizes that in Korea, the difference in auto-insurance claims made by victims of motor-traffic accidents from the use of traditional medicine when compared to conventional medicine, is not due to an actual service requirement, but to the opportunistic use by physicians who practice traditional medicine. As these costs are covered by insurance, the author speculates that these physicians may be prescribing unnecessary medications/procedures due the unawareness of patients and the power imbalance in the patient-physician relationship, leading to a physician-induced demand.

The author has used a sound decomposition analysis technique to test this hypothesis in a sample of Korean individuals; which has revealed that physicians' opportunistic behaviour is more prevalent among physicians who practice traditional medicine as opposed to those who practice conventional medicine.

While I congratulate the author on the efforts to unveil an interesting speculation, some comments for further improvement of this manuscript are mentioned below.

**Major comments**

1. The author has used a scientifically sound analysis using the modified Oaxaca-Blinder method by Chrenozhukov et al. (2013) to test his hypothesis. However, the justification for using it has not been put forward strongly.

    - It is recommended to rephrase the following sentence on page 9 accordingly: *"Chrenozhukov et al. (2013) method makes it possible to explain the difference in average medical use and the distribution of medical use" page 9* – please reword this sentence to clearly mention the advantage of using Chrenozhukov et al.'s method over the usual Oaxaca-Blinder method. This would give a stronger justification for the methodology used.

    - It may be also worthwhile to mention why this method was preferable to other economic decomposition methods.

2. The author is hypothesing that the difference in the two curves (use of herbal medicines versus conventional medicines by road traffic accident victims) may be due to the opportunistic behaviour of physicians - which is a structural effect - and has used decomposition analysis to test this.

- While it is commendable that the author has attempted to explain the concepts behind decomposition analysis (at the end of page 8 and beginning of page 9), it is suggested to rephrase this section in order to better explain the concepts behind decomposition analysis.
- The term 'structural effects' in relation to economics has not been clearly explained.
- Briefly explain what structural effects are, what they signify and to also explain the role of structural effects and endowment effect (covariates) in an outcome gap. This would be beneficial to a reader who is not well-versed in economic concepts.

3. The author has attributed the observed results to the opportunistic behavior of physicians who practice herbal medicine in the conclusions section (page 17, end of the second paragraph). The author has also mentioned this as an assumption in the study (page 8, first paragraph). It is not clearly mentioned how the results can be attributed to this behaviour, as there may be several structural causes for this difference.

4. It has been mentioned in the abstract that the observed results remained robust after controlling for patient characteristics and injury severity.

    - While the author has presented the covariates that were included in the model in Table 1, injury severity was not mentioned among these.
    - Was the variable named 'disability' a reflection of the severity of injury? If so, please mention this clearly.

5. Regarding Tables 1 and 2:

    - Means and standards deviations for categorical variables have been reported. Please clarify why. It is recommended to present categorical data as proportions.
    - Please describe what the variables that are used as covariates signify (e.g. '*college', 'labour income', 'disability', 'LOS'*– what do these variables mean?)
    - In Table 1, it is not clear what the variable "outpatient visit no" means. Please rename it as appropriate to indicate the number of outpatient visits.
    - It is recommended to present the income quintiles together under one variable, not presented in 5 separate variables as in the submitted manuscript.

6. The author has obtained data from the KHP and stated that only data on medical utilization covered by auto insurance were investigated (page 10). However, details on how many data points were analyzed are lacking. It is suggested to include these.

7. In the theoretical background, the author has mentioned certain conditions under which the study's assumptions would be valid (page 6, second paragraph). However, there is no mention of these conditions later on in the manuscript. Have these conditions been checked? If so, please include these in the manuscript.

8. The author has divided the difference in the conditional distribution by the difference in the observable distribution per quantile when reporting the results of the decomposition analysis (page 13 for Table 3 and page 15 for Table 4).

   - *Example: "Changes in the relationship of outpatient visits to the conditional distribution account for 8.96 visit differences (133%) of 6.697 visits, a 9.302 visit increase (102%) of 9.064, and 8.309 visits (115%) of 7.186 visit differences in the 7th, 8th, and 9th deciles, respectively." (page 13)*

   - It is suggested to mention that here the ratio of conditional to observable outcomes is being reported.
   - Furthermore, sentences such as the above need to be rephrased to clearly reflect results.

9. The last sentence of page 15 states "*In the overall quantile, the differences attributable to the determinants show negative values, but the differences attributable to structural factors are primarily positive.*".
   - It would be helpful to the reader if the significance of these positive and negative values is mentioned.

   This sentence is followed by the following interpretations on page 16:
   - "*The result shows that the LOS in herbal medicine hospitals is structurally more prolonged than in regular hospitals in the most quantile of LOS.*
   - *Hospitalization is usually highly severe, so it may show that patients with severe traffic accidents do not use herbal medicine often.*
   - *It may also indicate that patients who are severely injured in traffic accidents and are hospitalized through herbal medicine will likely stay longer in herbal medicine institutions than in regular hospitals.*"

- It would be clearer to the reader if each sentence can be explained in relation to results.

**Minor comments**

**1) Issues in the use of language –**

i. The two groups of physicians that are compared are not clearly described.
   - The terms ‘normal’, ‘usual’, ‘ordinary’ and ‘conventional’ are used to interchangeably to describe physicians who are practicing conventional medicine. Also, a consistent term needs to be used (instead of ‘herbal doctors’ and ‘oriental doctors’) to refer to physicians who practice traditional medicine.
   - It would be more formal to use a consistent term and describe what it denotes at the first instance where it’s used.

ii. It is recommended to avoid repetitions of the same concept.
   - For example, the sentence *“Because traffic accident victims are the recipients compensated for medical services from the auto insurance of the accident assaulter, medical services received by traffic accident victims are like a kind of in-kind service” (page 6)* has a similar meaning to the sentence prior to it *(“it can be said that auto insurance, which is compulsory insurance, is a system that provides in-kind medical services to victims of traffic accidents through the perpetrator's auto insurance”).*

iii. It is recommended to use one written voice/ style throughout the manuscript.
   - The first-person plural (we) and first-person singular (I) voice have been used interchangeably while some sections have been written using an impersonal voice (e.g. ‘this study aims to’).

iv. Please be mindful of the tense. As the study has already been carried out, please use a suitable tense to report it (present tense was used in some sections of the manuscript).

v. Language needs to be revisited throughout the manuscript.

**2) Labelling figures, tables and equations**

i. It is recommended to rename Figure 1 to indicate that three types of trends are shown (not only Korean herbal medicine claims). Also, describe what ‘normal medicine’ is.

ii. It is recommended to label the two graphs in Figure 2 as 'A' and 'B'. Refrain from referring to the graphs as 'left one' and 'right one' within the text (page 10, first paragraph second and third lines).

iii. Refrain from using abbreviations within tables and figures unless previously defined (e.g. '*k-medicine', 'LOS', 'b/w'*)

iv. It is recommended to describe what all variables in an equation signify (for example: in the equation for the net income *'Y'* on page 4, '*x'* has not been defined; the term $U_{11}$ has not been explained on page 5)

**3) Outline of the manuscript**

i. The last part of the first paragraph and the second paragraph on page 12 – (beginning from *"The sizable structural difference in the high quantile of outpatient visits…."* to the end of the second paragraph *"Therefore, this can explain the rapid increase in herbal medical costs in recent years."* – contains a reflection of the authors speculations based on the results observed.
   - These have been included in the Results section but should preferably be moved to the Discussion section as these are the authors speculations based on the results observed.
   - If the author wishes to keep these together, please label the section as Results and Discussion

ii. The same applies to the interpretation of results on Table 4, on page 15.

iii. Please try to divide the manuscript as recommended by the journal and arrange the subsections accordingly (https://journals.plos.org/plosone/s/submission-guidelines#loc-manuscript-organization)

   Example – the section titled Decomposition is your analysis method. Please label sections and subsections accordingly

iv. The second paragraph on page 5 – describes the outline of the manuscript. Please keep this only if necessary.

**4) Citations**

i. Please ensure that the citation style remains constant throughout the manuscript. The journal recommends using Vancouver style for citations.
   - Example – *McGuire (2002)* on page 4, *Jones (1996)* on page 5 etc. need to be changed to Vancouver style

ii. It would be more convincing to the reader if a reference could be added to support the following claims that doctors who practice herbal medicine manipulate the system:
   - *"In addition, due to the collective political pressure of herbal medicine doctors, they have been included in health insurance benefits since 2013." on page 6*
   - *"Herbal doctors are likely to take advantage of the legal status in which herbal medicine is not evaluated based on safety and effectiveness, unlike regular medical services." on page 7*

iii. Please re-check the bibliography (in Vancouver style the author's surname comes first, followed by initials)
