# Supplementary material for: Physician behavior for “invisible” treatment; Korean herbal medicine doctor’s treatment covered by auto insurance
Source: PLoS One. 2026 Jun 3;21(6):e0350512. doi: 10.1371/journal.pone.0350512 (PMC13232845; doi:10.1371/journal.pone.0350512)
Supplement: Attachment — Submitted filename: Rebuttal letter_LEE.docx [file pone.0350512.s001.docx]

**Response to Reviewers**

**Manuscript Title:** Physician behavior for “invisible” treatment; Korean herbal medicine doctor's treatment covered by auto insurance
**Manuscript Number:** *PONE-D-25-53640*

Dear Editor and Reviewers,

We sincerely thank the Academic Editor and the reviewers for their careful evaluation of our manuscript and for their constructive comments. We have carefully revised the manuscript in response to all feedback. Below, we provide a detailed point-by-point response. All revisions are highlighted in the *Revised Manuscript with Track Changes*, and the clean version has also been submitted as requested.

**Response to Editor**

**Comment 1 (Editor):**

*“The current version of the abstract is less informative. You are requested to revise it (especially the methods and results sections) before the review process can be further continued.”*

**Response:**
We appreciate the editor’s helpful guidance. In response, we have substantially revised the abstract to provide more precise and more detailed information on the study design, data source, empirical method, and main findings. Specifically, we now summarize the dataset used, the decomposition method applied, and the key quantitative results highlighting the structural differences in medical utilization between herbal and conventional medicine.

The updated abstract is as follows:

**Background**
This study examines the sharp increase in the use of Korean herbal medicine covered by auto insurance following traffic accidents, focusing on whether this trend reflects the opportunistic behavior of Korean herbal medicine doctors.

**Methods**
We extend the theoretical framework of physician-induced demand by incorporating differences in marginal costs between medical and herbal doctors. Using the Korea Health Panel data from 2017, we apply a recently developed decomposition method to compare utilization patterns between conventional and herbal medicine providers treating the same types of injuries.

**Results**
The analysis identifies significant structural differences in treatment behavior between herbal and medical doctors. At higher quantiles of medical utilization, herbal medicine shows disproportionately greater service provision, consistent with more substantial opportunistic incentives. Changes in the relationship between outpatient visits and structural differences account for 8.96 visit differences (133%) of 6.697 visits, a 9.302 visit increase (102%) of 9.064 visits, and 8.309 visit differences (115%) of 7.186 visits in the 7th, 8th, and 9th deciles, respectively. Changes in the relationship of hospital stay to the structural differences account for seven-day differences (442%) of a 1.5-day increase in the 7th decile, two days (300%) of a 1-day decrease in the 8th decile, and 8.842 days (186%) of a 4.75-day increase in the 9th decile. These results remain robust after controlling for patient characteristics and injury severity.

**Conclusions**
Findings suggest that herbal medicine services under auto insurance are more structurally driven by provider behavior than patient need, implying a need for closer scrutiny of reimbursement and review procedures to mitigate opportunistic practice.
