# Supplementary material for: Physician behavior for “invisible” treatment; Korean herbal medicine doctor’s treatment covered by auto insurance
Source: PLoS One. 2026 Jun 3;21(6):e0350512. doi: 10.1371/journal.pone.0350512 (PMC13232845; doi:10.1371/journal.pone.0350512)
Supplement: Attachment — Submitted filename: Response to Reviewers_2ndRevision.docx [file pone.0350512.s003.docx]

**[Response to Reviewer #1]**

**Major Comment 1: Justification for the Chernozhukov et al. (2013) method.**

**Response:** We thank the reviewer for this insightful suggestion. We have revised the manuscript (Page 8) to clearly articulate the advantages of the **Chernozhukov et al. (2013)** method over the traditional Oaxaca-Blinder approach. Specifically, we now emphasize that while the traditional method only captures differences in the **mean**, the functional decomposition method allows us to analyze the **entire distribution (quantiles)**. This is crucial for our hypothesis, as opportunistic provider behavior—represented here as a **structural effect**—is more likely to be prevalent in the higher deciles of healthcare utilization. We have also clarified the roles of **endowment effects** and **structural effects** to make the methodology more accessible to a broader audience.

**Major Comment 2: Clarifying “Structural Effects.”**

**Response:** We have added a brief explanation in the Methods section. We now clarify that **Endowment Effects** refer to differences in patient characteristics (e.g., age, education, income), while **Structural Effects** represent the 'unexplained' gap—which, in this study, we attribute to the differing incentive structures and behaviors of Korean Herbal Medicine (KHM) vs. Conventional Medicine (CM) providers.

**Major Comment 3: Clarifying “Opportunistic behavior of the physician.”**

**Response:** We now frame the structural differences as being **indicative of supply-side incentives** rather than definitive proof of opportunistic behavior. We have also explicitly acknowledged the **limitations of causal inference** in our observational study design.

**Major Comment 4: Clarifying “Injury severity.”**

**Response:** The variable named ‘disability’ is not a reflection of the severity of injury. We don’t have any information on the severity of injury in the data. We added an explanation of the ‘disability’ definition in the Variable section. We have also removed the injury severity from the manuscript to avoid confusion.

**Major Comment 5: Regarding Table 1 and 2**

**Response:** We explicitly define "LOS" (Length of Stay), "Income Quintiles," and rename "outpatient visit no" to "**Number of Outpatient Visits.**" We added an explanation of the ‘disability’ definition in the Variable section. ‘Disability’ dummy indicates whether the respondent is classified as having a disability by the government.

**Major Comment 6: Regarding the observation number of the data.**

**Response:** We have added details on how many observations are used for this study in the data section. For the outpatient covered by auto insurance, 187 observations out of 301,540 are observed, and for the inpatient covered by auto insurance, 95 observations out of 3521 are observed in the 2017 KHP.

**Major Comment 7: Certain conditions in the theoretical background.**

**Response:** We have added a statement that our empirical results indicate the marginal utility of KHM doctors in inducing demand may exceed that of CM doctors, consistent with the theoretical background in the manuscript.

**Major Comment 8 & 9: Reporting the ratio of conditional to observable outcomes.**

**Response:** We have revised the reporting of our results on Pages 13 and 15 to enhance clarity. We now explicitly state that we are reporting the **ratio of the structural (conditional) effect to the total observed difference**. We have also rephrased the results to clearly reflect that **positive structural values** signify provider-side drivers of utilization, while **negative endowment values** indicate that patient characteristics do not explain the observed gap. This clarification ensures that the significance of these values is immediately accessible to the reader.

**Minor Comments: Use of language, Labelling figures, tables and equations,**

**Response:** We consistently use **Conventional Medicine (CM) doctors** and **Korean Herbal Medicine (KHM) doctors**. We also changed the labeling accordingly. We have switched entirely to an **impersonal voice** (e.g., "This study finds...") or to a consistent "We" if the journal allows it. We have converted all citations to **Vancouver Style** as required by PLOS ONE.

**[Response to Reviewer #2]**

**Comment 1: Absence of a separate Methods section.**

**Response:** We sincerely apologize for the lack of clarity. Following your suggestion, we have created a dedicated **'Methods'** section which consolidates empirical methods, data and variables previously dispersed throughout the manuscript.

**Comment 2: Study design not explicitly stated.**

**Response:** We have now explicitly identified the study as a **cross-sectional secondary data analysis** in both the Abstract and the new Methods section.

**Comment 3: Incomplete description of participants.**

**Response:** We have added details on how many observations are used for this study in the data section. For the outpatient covered by auto insurance, 187 observations out of 301,540 are observed, and for the inpatient covered by auto insurance, 95 observations out of 3521 are observed in the 2017 KHP.

**Comment 4: Insufficient discussion of bias and confounding.**

**Response:** We have expanded the Limitations section to include a discussion on the **lack of clinical severity measures** and the potential for **unmeasured confounding**.

**Comment 5: Unclear variable definitions.**

**Response:** We have added a variable section and explained the variables used in the study.

**Comment 6: Overinterpretation of findings.**

**Response:** We have carefully revised the Results and Conclusions sections to soften our claims. We now frame the findings as **"indicative of structural incentives"** rather than "direct proof of opportunistic behavior," acknowledging the constraints of observational research.

**Comment 7: Limited discussion of study limitations.**

**Response:** We have expanded the Limitations section to include a discussion on the **lack of clinical severity measures** and the potential for **unmeasured confounding**.

**Comment 8: Inconsistent terminology (Normal, Usual, etc.).**

**Response:** We sincerely apologize for the confusion. We have standardized all terms to **"Conventional Medicine (CM) doctors"** and **“Korean Herbal Medicine (KHM) doctors”** throughout the revised manuscript.

**Comment 9: Use of first-person narrative**

**Response:** We have switched entirely to an **impersonal voice** (e.g., "This study finds...") or a consistent "We" if the journal allows it.
